# Characterization of sacral chordoma and differential diagnosis from other sacral malignancy using [^18^F]FDG PET/CT

**DOI:** 10.1097/MD.0000000000037678

**Published:** 2024-04-05

**Authors:** Dong Yun Lee, Yong-il Kim, Jin-Sook Ryu, Wanlim Kim

**Affiliations:** a Department of Nuclear Medicine, Asan Medical Center, University of Ulsan College of Medicine, Seoul, Korea; b Department of Orthopedic Surgery, Asan Medical Center, University of Ulsan College of Medicine, Seoul, Korea.

**Keywords:** chordoma, fluorodeoxyglucose F18, malignant bone tumor, positron emission tomography computed tomography, sacral tumor, sensitivity and specificity

## Abstract

2-Deoxy-2-[^18^F]fluoro-D-glucose ([^18^F]FDG) positron emission tomography (PET)/computed tomography (CT) is known to be a helpful imaging modality for sacral chordoma, but its detailed characteristics have not been fully described. The purpose of our study was to identify the [^18^F]FDG PET/CT imaging characteristics of sacral chordoma and compare them with other sacral malignancy. This retrospective study included patients who underwent [^18^F]FDG PET/CT because of a mass involving the sacrum. Investigated visual findings included visual score and distribution, and semiquantitative parameters measured included standardized uptake values (SUVmax, SUVpeak, SUVmean), tumor-to-liver ratio (TLR), metabolic tumor volume (MTV), total lesion glycolysis (TLG), and tumor size. Comparison studies and receiver operating characteristics (ROC) curve analysis were performed to differentiate between sacral chordoma and other sacral malignancy. Ten patients with sacral chordoma were finally included (M:F = 6:4, median age = 67 yr). On [^18^F]FDG PET/CT, sacral chordomas presented as a mass with minimal–moderate uptake with a usually heterogenous distribution. Compared with 12 patients with other sacral malignancies (M:F = 4:8, median age 42 yr), sacral chordoma showed a significantly lower TLR (median value 2.1 vs 6.3, *P* = .021). In ROC curve analysis, TLR showed the largest area under the curve (AUC) of 0.79 (cutoff ≤ 4.0; sensitivity 100.0%, specificity 58.3%; *P* = .004), and SUVmax showed the second largest AUC of 0.73 (cutoff ≤ 6.9; sensitivity 80.0%, specificity 66.7%; *P* = .034). [^18^F]FDG PET/CT of sacral chordoma showed minimal–moderate uptake. The TLR of [^18^F]FDG PET/CT was significantly lower than that of other sacral malignancy and was the most useful parameter for differentiating sacral chordoma, with the largest AUC. SUVmax could be another helpful semiquantitative parameter.

## 1. Introduction

Sacral tumors account for 1% to 4% of spinal tumors and include a wide range of primary and secondary tumors.^[1]^ Radiographic changes caused by sacral tumors are known to be slight.^[2]^ Therefore, advanced imaging techniques such as computed tomography (CT) and magnetic resonance imaging (MRI) play important roles in accurate lesion characterization and management guidance.^[3]^

Chordomas are generally rare tumors that make up 2% to 4% of all primary malignant bone tumors.^[4]^ However, about 50% to 60% of chordomas occur in the sacrococcygeal region, and they are the most common primary malignant sacral tumor, constituting about 40% to 50% of all primary sacral tumors.^[5]^ Chordomas have a slight male predominance and mainly manifest at ages between 40 and 70 years.^[6]^ They are slow-growing, locally aggressive tumors accompanied with pain symptoms associated with large masses.^[7]^ On CT, sacral chordomas appear as lobulated expansile masses, presenting with 30% to 70% calcification.^[8]^ On MRI, sacral chordoma usually shows heterogenous high signal on T2-weighted imaging and scattered high signal on T1-weighted imaging.^[9]^

2-Deoxy-2-[^18^F]fluoro-D-glucose ([^18^F]FDG) positron emission tomography (PET)/CT is increasingly being used as a noninvasive imaging modality for accurate staging and assessment of recurrent/metastatic sacral tumors.^[10,11]^ Unlike other malignant tumors, [^18^F]FDG uptake of chordomas is known to be mild or moderate.^[12]^ Nevertheless, only a few reports have described the detailed visual, metabolic, and volumetric characteristics of sacral chordomas on [^18^F]FDG PET/CT.^[13,14]^ The aim of this study was to investigate the [^18^F]FDG PET/CT imaging characteristics of pathologically proven sacral chordomas and to compare them with other sacral malignancy.

## 2. Patients and methods

### 2.1. Study design and patients

**Figure 1.**
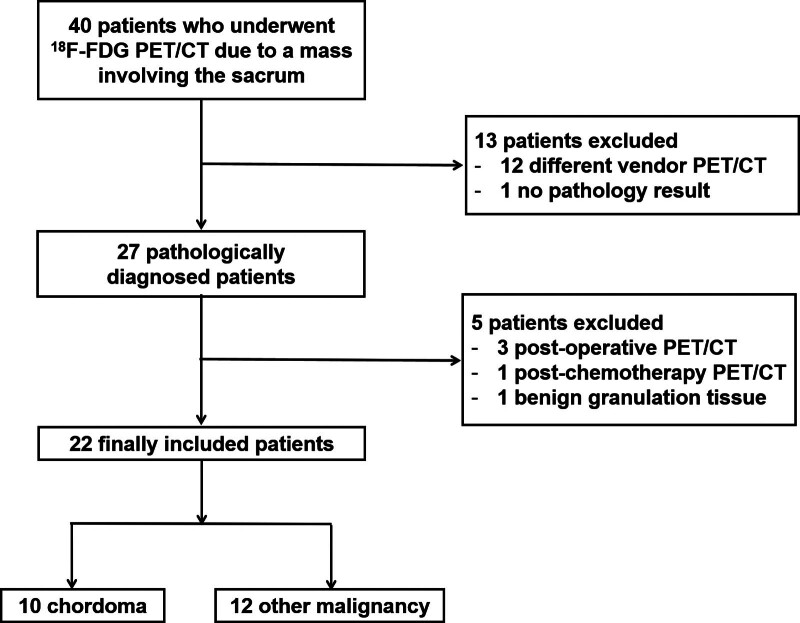
Flowchart of patient selection.

We retrospectively reviewed the medical records of 40 patients who underwent [^18^F]FDG PET/CT because of a mass involving the sacrum between January 2013 and November 2023. Among them, patients who underwent [^18^F]FDG PET/CT imaging at our institution within the 6 months before pathologic confirmation were finally included (Fig. 1). Our institutional review board approved the study design and an informed patient consent waiver (no. 2023-1539).

### 2.2. [^18^F]FDG PET/CT imaging protocol

All of the included patients fasted for at least 6 hours before [^18^F]FDG PET/CT image acquisition, and their venous blood glucose levels were maintained below 150 mg/dL. [^18^F]FDG was intravenously administered at 5.2 MBq/kg and PET/CT image acquisition was performed 1 hour after this administration (Discovery PET/CT 690, 690 Elite, 710; GE Healthcare, Milwaukee, WI). First, the CT images were acquired from the skull base to the upper thigh in the case of torso PET/CT, or from the vertex to the sole in the case of whole-body PET/CT. These acquisitions used the following parameters: 120 kVp, automatic mA, 40 mm collimation, and 3.75 mm slice thickness. PET images of the same area were acquired after the CT scan using a 3-dimensional mode with 2 minutes per bed position, spanning from 6 beds (torso) to 16 beds (whole-body). Attenuation correction and image reconstruction were performed using the 3-dimensional ordered subset expectation maximization method with time-of-flight (TOF) and point-spread-function algorithms (192 × 192 matrix, 4 iterations, 18 subsets, and 4 mm post-smoothing).

### 2.3. [^18^F]FDG PET/CT image analysis

[^18^F]FDG PET/CT images were reviewed using Mirada DBx workstation (version 1.2.0.59; Mirada Medical Ltd., Oxford, UK) by a board-certified nuclear medicine physician who was blinded to the clinical data. First, the PET/CT images were visually assessed and sacral masses were categorized according to their [^18^F]FDG PET/CT visual score, distribution, and shape. The visual scores were defined as follows: score 1, lower or equal to mediastinal uptake (minimal); score 2, greater than mediastinal uptake but lower or equal to liver uptake (mild); score 3, slightly greater than liver uptake (moderate); grade 4, much greater than liver uptake (intense). The distribution of uptake was defined as heterogeneous (uneven uptake) or homogenous (uniform uptake). All visual findings were evaluated using PET, CT, and PET/CT fusion images.

Next, sacral masses on the [^18^F]FDG PET/CT images were semiquantitatively analyzed. A volume-of-interest large enough to cover the sacral mass was delineated. First, the maximum standardized uptake value (SUVmax), peak SUV (SUVpeak), and mean SUV (SUVmean) of the sacral mass were extracted. Second, the tumor-to-liver ratio (TLR) was calculated as the SUVmax of the sacral mass divided by the SUVmean of more than 10 cm^3^ of the right lower lobe of the liver.^[15,16]^ Third, the volumetric parameter of metabolic tumor volume (MTV) was measured by applying a threshold of 50% of the SUVmax within the volume-of-interest, and total lesion glycolysis (TLG) was calculated as MTV multiplied by SUVmean.^[17]^ Finally, the tumor size of the lesion was measured on the combined axial CT image.

### 2.4. Statistical analysis

Patient age was expressed as median with range. The statistical significances of gender, age, and visual findings for differentiating between sacral chordoma and other sacral malignancy were analyzed using Pearson’s chi-square test and Fischer’s exact test. Semiquantitative [^18^F]FDG PET/CT parameters were analyzed using the Mann–Whitney *U* test. Receiver operating characteristics (ROC) curve analysis was performed to evaluate the diagnostic performance of sacral chordoma with area under the curve (AUC) values and their 95% confidence intervals. Optimal cutoff values were defined as the exploratory cutoff values with the highest accuracy according to Youden’s Index, and sensitivity and specificity were assessed according to the optimal cutoff values. Finally, the subgroup analysis comparing sacral chordoma and diffuse large B cell lymphoma was performed using the Mann–Whitney *U* test. A *P*-value <.05 was considered statistically significant. Statistical analyses were performed using SPSS software version 18.0 (SPSS, Chicago, IL) or MedCalc version 19.2.3 (MedCalc Software Ltd., Ostend, Belgium).

## 3. Results

### 3.1. Patient profiles and sacral chordoma characteristics

A total of 22 patients (10 males and 12 females) were finally included in the analysis (Fig. 1). The median age of the patients was 60 years (range 8–90). Among them, 10 patients had sacral chordoma and 12 patients had other sacral malignancy. The pathologies of the other sacral malignancies were diffuse large B cell lymphoma in 2 patients, chondrosarcoma in 2 patients, mucinous adenocarcinoma in 2 patients, yolk sac tumor in 2 patients, Ewing sarcoma in 1 patient, osteosarcoma in 1 patient, rhabdoid tumor in 1 patient, and esophageal cancer metastasis in 1 patient (Table 1). Sacral chordomas had a mass size of 2.7 to 12.5 cm, with SUVmax of 2.0 to 7.9 and TLR of 0.7 to 4.0 (Table 2). Results summary for other sacral malignancies is provided in Table S1, Supplemental Digital Content, http://links.lww.com/MD/M91.

**Table 1:** Patient profiles.

Characteristics	All sacral malignancy	Sacral chordoma	Other sacral malignancy	*P*-value
Patient no.	22	10	12	
Gender (male:female)	10:12	6:4	4:8	.391
Age (yr)	60 (2–90)	67 (42–90)	42 (2–88)	.060
Pathology				
Sacral chordoma	10	10		
Diffuse large B cell lymphoma	2		2	
Chondrosarcoma	2		2	
Mucinous adenocarcinoma	2		2	
Yolk sac tumor	2		2	
Ewing sarcoma	1		1	
Osteosarcoma	1		1	
Rhabdoid tumor	1		1	
Esophageal cancer metastasis	1		1	
Disease pattern				
Primary	19	10	9	
Recurrence	2		2	
Metastasis	1		1	

**Table 2:** ^18^F-FDG PET/CT characteristics of sacral chordoma.

No.	Gender	Age	Visual score	Distribution	Tumor size (cm)	SUVmax	SUVpeak	SUVmean	TLR	MTV (cm^3^)	TLG
1	Female	42	3	Heterogenous	5.2	3.9	2.7	2.4	1.7	15.8	37.7
2	Female	90	1	Homogenous	6.7	2.0	1.7	1.4	0.7	10.8	15.5
3	Male	85	3	Homogenous	3.2	6.7	5.2	4.4	2.7	7.3	31.9
4	Male	58	3	Heterogenous	8.0	5.1	4.2	3.1	2.2	41.9	129.6
5	Male	74	3	Heterogenous	12.5	7.9	5.9	4.7	4.0	62.1	290.4
6	Female	81	3	Heterogenous	5.2	5.6	3.9	3.4	1.9	23.5	79.2
7	Female	65	3	Heterogenous	10.7	6.3	4.1	3.6	2.5	17.1	61.3
8	Male	58	3	Heterogenous	2.7	4.8	3.5	3.2	2.0	3.4	10.9
9	Male	57	3	Heterogenous	6.6	7.7	5.9	5.0	3.0	30.5	151.2
10	Male	68	2	Heterogenous	5.1	4.3	2.9	2.6	1.7	18.4	48.3

MTV = metabolic tumor volume, SUV = standardized uptake value, TLG = total lesion glycolysis, TLR = tumor-to-liver ratio.

### 3.2. Visual findings

Most of the sacral malignancies demonstrated uptake with a score of 3 or more (18/22, 81.8%) and a heterogenous distribution (18/22, 81.8%). No sacral chordoma showed an uptake score of 4 and no other sacral malignancy showed an uptake score of 0. The distribution of sacral chordoma was usually heterogenous (8/10, 80.0%). No significant difference in visual findings was found between sacral chordoma and other sacral malignancy (Table 3).

**Table 3:** Comparison of ^18^F-FDG PET/CT visual findings between sacral chordoma and other sacral malignancy.

Visual findings	All sacral malignancy (n = 22)	Sacral chordoma (n = 10)	Other sacral malignancy (n = 12)	*P*-value
Visual score				.140
Score 4	4	0	4	
Score 3	14	8	6	
Score 2	3	1	2	
Score 1	1	1	0	
Distribution				1.000
Heterogenous	18	8	10	
Homogenous	4	2	2	

### 3.3. Semiquantitative analysis

**Figure 2.**
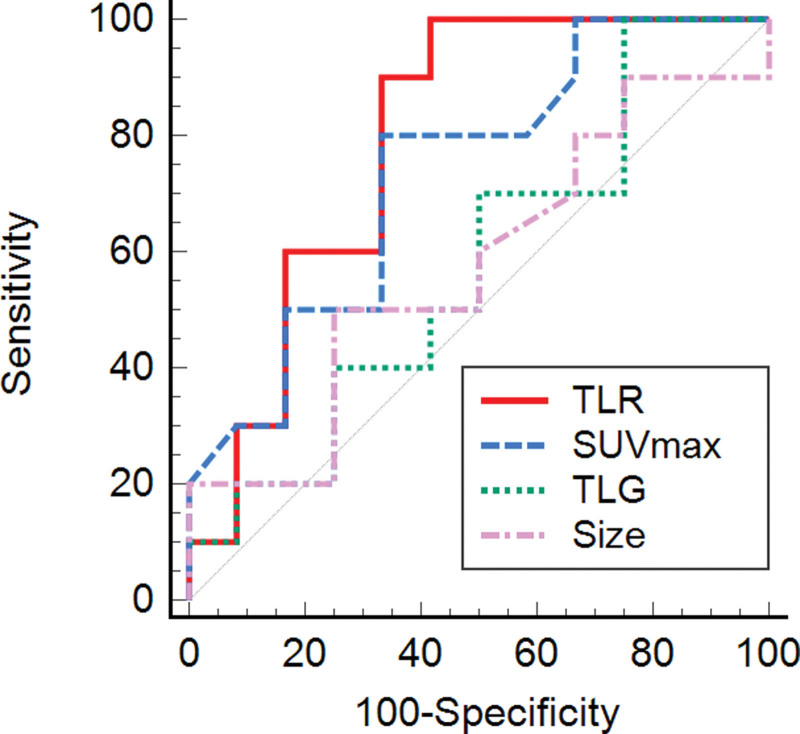
Receiver operating characteristics (ROC) curve analysis of semiquantitative parameters. In the ROC curve analysis, tumor-to-liver ratio (TLR) showed the largest area under the curve of 0.79 (*P* = .004) for diagnosis of sacral chordoma. The sensitivity and specificity of TLR (optimal cutoff of ≤4.0 to ≤4.0) were 100.0% and 58.3%, respectively. ROC = Receiver operating characteristics, TLR = tumor-to-liver ratio.

**Figure 3.**
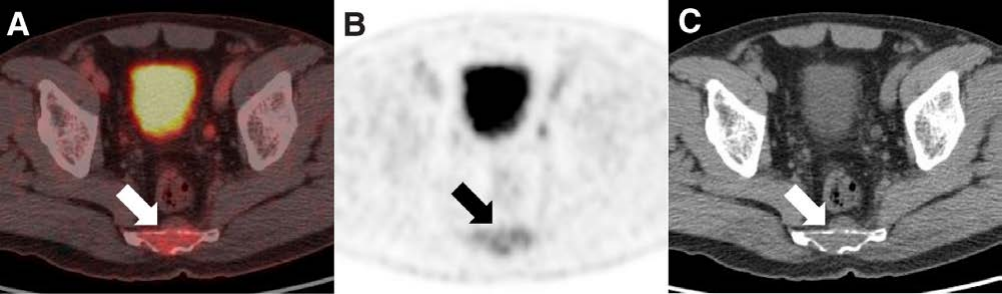
A 68-year-old man who had sacral chordoma. Axial (A) PET/CT, (B) PET, and (C) CT images show a 6.7 cm mass in the sacrum with a visual score of 2 and heterogenous uptake. The tumor-to-liver ratio (TLR), maximum standardized uptake value (SUVmax), and total lesion glycolysis (TLG) were 1.7, 5.1, and 48.3, respectively. The mass was proven as sacral chordoma after excision. CT = computed tomography, PET = positron emission tomography, TLR = tumor-to-liver ratio.

**Figure 4.**
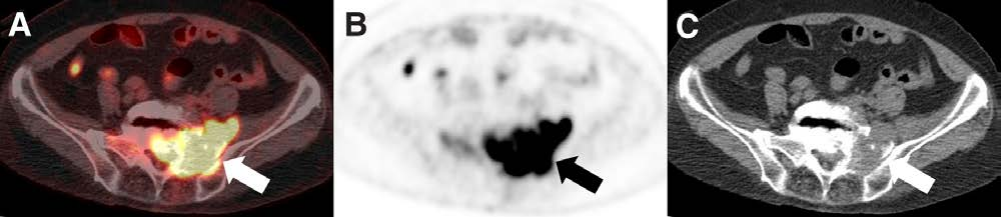
A 78-year-old woman who had sacral diffuse large B cell lymphoma (DLBL). Axial (A) PET/CT, (B) PET, and (C) CT images show a 6.7 cm mass in the sacrum with a visual score of 4 and homogenous uptake. The tumor-to-liver ratio (TLR), maximum standardized uptake value (SUVmax), and total lesion glycolysis (TLG) were 15.1, 33.7, and 401.2, respectively. The mass was proven as sacral DLBL after needle biopsy. DLBL = diffuse large B cell lymphoma, CT = computed tomography, PET = positron emission tomography, TLR = tumor-to-liver ratio.

The only semiquantitative [^18^F]FDG PET/CT parameter showing a significant difference between sacral chordoma and other sacral malignancy was TLR (*P* = .021; Table 4). In the ROC curve analysis, TLR showed the largest AUC (AUC = 0.79, cutoff ≤ 4.0; *P* = .004) and SUVmax showed the second largest AUC (AUC = 0.73, cutoff ≤ 6.9; *P* = .034) (Fig. 2). The sensitivity and specificity of TLR using a cutoff value of 4.0 were 100.0% and 58.3%, respectively (Figs. 3 and 4). Other semiquantitative parameters showed no significance (Table 5).

**Table 4:** Comparison of ^18^F-FDG PET/CT semiquantitative parameters between sacral chordoma and other sacral malignancy.

Parameters	All sacral malignancy (n = 22)	Sacral chordoma (n = 10)	Other sacral malignancy (n = 12)	*P*-value
Tumor size (cm)	6.5 (2.7–12.5)	5.9 (2.7–12.5)	6.5 (3.8–11.9)	.582
SUVmax	6.5 (2.3–33.7)	5.4 (2.3–7.9)	7.4 (4.3–33.7)	.069
SUVpeak	4.4 (1.7–21.5)	4.0 (1.7–5.9)	5.2 (2.8–21.5)	.159
SUVmean	3.9 (1.4–19.0)	3.3 (1.4–5.0)	4.4 (2.6–19.0)	.093
TLR	2.6 (0.7–15.1)	2.1 (0.7–4.0)	6.3 (1.4–15.1)	.021*
MTV (cm^3^)	17.8 (3.4–88.8)	17.8 (3.4–62.1)	17.8 (4.0–88.8)	.582
TLG	57.4 (10.9–676.0)	54.8 (10.9–290.4)	66.6 (12.3–676.0)	.771

MTV = metabolic tumor volume, SUV = standardized uptake value, TLG = total lesion glycolysis, TLR = tumor-to-liver ratio.

* *P* < .05.

**Table 5:** ROC curve analysis of ^18^F-FDG PET/CT semiquantitative parameters for diagnosis of sacral chordoma.

Parameters	Sensitivity (%)	Specificity (%)	AUC	cutoff	*P*-value
Tumor size (cm)	50.0	75.0	0.58 (0.35–0.78)	≤5.2	.566
SUVmax	80.0	66.7	0.73 (0.50–0.90)	≤6.9	.034*
SUVpeak	70.0	66.7	0.68 (0.45–0.86)	≤4.2	.119
SUVmean	70.0	66.7	0.71 (0.48–0.88)	≤3.6	.059
TLR	100.0	58.3	0.79 (0.57–0.93)	≤4.0	.004*
MTV (cm^3^)	90.0	33.3	0.54 (0.32–0.75)	>6.9	.750
TLG	100.0	25.0	0.58 (0.35–0.78)	≤290.4	.559

AUC = area under the curve, MTV = metabolic tumor volume, ROC = receiver operating characteristics, SUV = standardized uptake value, TLG = total lesion, TLR = tumor-to-liver ratio.

* *P* < .05.

### 3.4. Subgroup analysis

We performed subgroup analysis comparing sacral chordoma and another single histology of sacral malignancy (diffuse large B cell lymphoma). TLR (*P* = .031), SUVmax (*P* = .030), SUVpeak (*P* = .031), and SUVmean (*P* = .030) were significantly lower in sacral chordoma than in diffuse large B cell lymphoma (Table S2, Supplemental Digital Content, http://links.lww.com/MD/M92).

## 4. Discussion

In our study, sacral chordomas revealed a visual score of 1 to 3 and had a mainly heterogenous uptake on [^18^F]FDG PET/CT. Among the semiquantitative [^18^F]FDG PET/CT parameters studied, TLR demonstrated a significantly lower value in sacral chordoma than in other sacral malignancy. ROC curve analysis showed that TLR and SUVmax were significant parameters for differentiating between sacral chordoma and other sacral malignancy, with TLR having the highest AUC. Therefore, we conclude that TLR is the best [^18^F]FDG PET/CT parameter for characterizing sacral chordomas. As far as we are aware, our study is the first to compare [^18^F]FDG PET/CT between sacral chordoma and other sacral malignancies.

We found that sacral chordomas demonstrated variable minimal to moderate uptake. Ishibashi et al and Thorn et al reported that chordomas show low to moderate uptake, which is in accord with our study.^[12,18]^ Previous case reports and small series studies reported SUVmax of between 2.1 and 5.8 for chordomas, values that are similar to ours (SUVmax of 2.0–7.9).^[14,19]^ Chordoma’s slow-growing osteolytic characteristics and low cellularity with abundant mucin histology may explain the relatively low [^18^F]FDG uptake.^[20]^ Although overlap exists between sacral chordoma and other sacral malignancies, visual findings of intense uptake by a mass might be a simple way to exclude sacral chordoma. Olson et al reported that chordomas demonstrate heterogenous, moderate [^18^F]FDG uptake.^[21]^ In case reports, Lin et al and Park et al reported sacral chordoma with heterogeneously increased [^18^F]FDG uptake, which is in accord with our findings.^[13,14]^ Such heterogenous uptake may be partly due to the presence of hemorrhage and protein content.^[2]^

Our comparison of semiquantitative parameters revealed TLR to be the best parameter for differentiating between sacral chordoma and other sacral malignancy, with it showing the largest AUC in the ROC curve analysis. Although SUVmax is the most widely used semiquantitative PET/CT parameter, variations across PET/CT scanners, protocols, and patient factors make it hard to generalize results.^[22,23]^ As an alternative parameter, TLR may show lower interscan variability because of the reference normalization (usually using normal liver uptake), which results in better reproducibility than SUVmax.^[24,25]^ In this regard, our study results imply that TLR could be widely applied. However, future comparison studies between TLR and SUVmax should be conducted with more patients because both TLR and SUVmax (the second largest AUC) showed significant results. In addition, we compared sacral chordoma and diffuse large B cell lymphoma in a subgroup analysis excluding recurrence/metastasis and limited to a single histology. This subgroup analysis showed that TLR and SUVmax can be semiquantitative parameters to characterize sacral chordoma irrespective of disease pattern and to differentiate sacral malignancies with high [^18^F]FDG uptake. In a case report, [^18^F]FDG uptake of recurrent chordoma was higher than that of primary chordoma, which indirectly supports our study results.^[26]^

This study has some limitations. As this investigation is a retrospective single center study, the possibility of referral bias cannot be ruled out. In addition, benign sacral tumors, such as giant cell tumor, were not included in our study, and the usefulness of [^18^F]FDG PET/CT for differentiating between sacral chordoma and benign sacral tumor needs to be studied.^[27,28]^ Finally, as the number of sacral chordoma patients was relatively small, the prognostic value of [^18^F]FDG PET/CT could not be assessed. A multicenter prospective study is required in the future.

## 5. Conclusions

In conclusion, our study found distinctive features of sacral chordoma using [^18^F]FDG PET/CT. Sacral chordoma showed variable minimal to moderate uptake, and TLR on [^18^F]FDG PET/CT could provide the best information for differentiating between sacral chordoma and other sacral malignancy. The SUVmax of [^18^F]FDG PET/CT could also be another useful parameter.

## Author contributions

**Conceptualization:** Yong-il Kim.

**Data curation:** Wanlim Kim.

**Formal analysis:** Dong Yun Lee, Yong-il Kim.

**Methodology:** Yong-il Kim, Jin-Sook Ryu.

**Resources:** Wanlim Kim.

**Writing – original draft:** Dong Yun Lee, Yong-il Kim, Wanlim Kim.

**Writing – review & editing:** Jin-Sook Ryu.

## Supplementary Material




