# Supplementary material for: Characterization of sacral chordoma and differential diagnosis from other sacral malignancy using [18F]FDG PET/CT
Source: Medicine (Baltimore). 2024 Apr 5;103(14):e37678. doi: 10.1097/MD.0000000000037678 (PMC10994510; doi:10.1097/MD.0000000000037678)
Supplement: Supplementary file 1 [file medi-103-e37678-s001.docx]

**Supplementary Table 1.** Characteristics of other sacral malignancies

| No. | Gender | Age  (yrs) | Disease | Disease pattern | Visual  score | Distribution | Tumor  size (cm) | SUVmax | SUVpeak | SUVmean | TLR | MTV  (cm^3^) | TLG |
| --- | --- | --- | --- | --- | --- | --- | --- | --- | --- | --- | --- | --- | --- |
| 1 | Female | 88 | DLBL | Primary | 4 | Homogenous | 6.7 | 33.7 | 21.5 | 19.0 | 15.1 | 21.1 | 401.2 |
| 2 | Female | 26 | DLBL | Primary | 4 | Heterogenous | 3.8 | 18.7 | 11.7 | 11.5 | 7.0 | 4.1 | 46.7 |
| 3 | Female | 62 | Chondrosarcoma | Primary | 3 | Heterogenous | 10.9 | 5.5 | 2.8 | 3.1 | 2.3 | 4.0 | 12.3 |
| 4 | Male | 72 | Chondrosarcoma | Primary | 2 | Heterogenous | 11.4 | 4.4 | 3.1 | 2.6 | 1.8 | 38.2 | 98.1 |
| 5 | Female | 66 | Mucinous adenocarcinoma | Recurrence | 2 | Heterogenous | 5.9 | 4.3 | 3.3 | 2.7 | 1.4 | 29.5 | 79.6 |
| 6 | Male | 57 | Mucinous adenocarcinoma | Primary | 3 | Heterogenous | 11.9 | 6.9 | 5.3 | 4.3 | 3.1 | 12.4 | 53.5 |
| 7 | Female | 3 | Yolk sac tumor | Recurrence | 3 | Heterogenous | 4.8 | 7.7 | 5.8 | 5.0 | 6.6 | 23.1 | 114.8 |
| 8 | Female | 2 | Yolk sac tumor | Primary | 3 | Heterogenous | 6.3 | 7.6 | 4.5 | 4.2 | 7.9 | 4.5 | 19.2 |
| 9 | Female | 17 | Ewing sarcoma | Primary | 4 | Heterogenous | 9.9 | 11.9 | 10.0 | 7.6 | 7.9 | 88.8 | 676.0 |
| 10 | Male | 20 | Osteosarcoma | Primary | 3 | Heterogenous | 6.7 | 5.5 | 3.8 | 3.3 | 2.4 | 14.4 | 46.6 |
| 11 | Female | 8 | Rhabdoid tumor | Primary | 3 | Heterogenous | 4.3 | 7.2 | 5.0 | 4.5 | 5.9 | 6.9 | 31.0 |
| 12 | Male | 70 | Esophageal cancer metastasis | Metastasis | 4 | Homogenous | 5.5 | 17.2 | 14.0 | 11.6 | 7.0 | 40.9 | 473.5 |

DLBL = Diffuse large B cell lymphoma; SUV = standardized uptake value; TLR = tumor-to-liver ratio; MTV = metabolic tumor volume; TLG = total lesion glycolysis
