# Supplementary material for: Characterization of sacral chordoma and differential diagnosis from other sacral malignancy using [18F]FDG PET/CT
Source: Medicine (Baltimore). 2024 Apr 5;103(14):e37678. doi: 10.1097/MD.0000000000037678 (PMC10994510; doi:10.1097/MD.0000000000037678)
Supplement: Supplementary file 2 [file medi-103-e37678-s002.docx]

**Supplementary Table 2.** Subgroup comparison between sacral chordoma and diffuse large B cell lymphoma

| Parameters | Sacral chordoma (n = 10) | Diffuse large B cell lymphoma (n = 2) | *P*-value |
| --- | --- | --- | --- |
| Tumor size (cm) | 5.9 (2.7–12.5) | 5.3 (3.8–6.7) | 0.746 |
| SUVmax | 5.4 (2.3–7.9) | 26.2 (18.7–33.7) | 0.030* |
| SUVpeak | 4.0 (1.7–5.9) | 16.6 (11.7–21.5) | 0.031* |
| SUVmean | 3.3 (1.4–5.0) | 15.3 (11.5–19.0) | 0.030* |
| TLR | 2.1 (0.7–4.0) | 11.1 (7.0–15.1) | 0.031* |
| MTV (cm3) | 17.8 (3.4–62.1) | 12.6 (4.1–21.1) | 0.606 |
| TLG | 54.8 (10.9–290.4) | 224.0 (46.7–401.2) | 0.485 |

SUV = standardized uptake value; TLR = tumor-to-liver ratio; MTV = metabolic tumor volume; TLG = total lesion glycolysis

**P* < 0.05
